# Mapping Eye-Tracking Research in Human–Computer Interaction: A Science-Mapping and Content-Analysis Study

**DOI:** 10.3390/jemr19010023

**Published:** 2026-02-12

**Authors:** Adem Korkmaz

**Affiliations:** Department of Computer Technologies, Gönen Vocational School, Bandırma Onyedi Eylül University, Bandırma 10200, Türkiye; ademkorkmaz@bandirma.edu.tr

**Keywords:** eye tracking, human–computer interaction (HCI), extended reality (XR), gaze estimation, cognitive load, multimodal interaction, bibliometric analysis, content analysis

## Abstract

Eye tracking has become a central method in human–computer interaction (HCI), supported by advances in sensing technologies and AI-based gaze analysis. Despite this rapid growth, a comprehensive and up-to-date overview of eye-tracking research across the broader HCI landscape remains lacking. This study combines records from Web of Science (WoS) and Scopus to analyse 1033 publications on eye tracking in HCI published between 2020 and 2025. After merging and deduplicating the datasets, we conducted bibliometric network analyses (keyword co-occurrence, co-citation, co-authorship, and source mapping) using VOSviewer and performed a qualitative content analysis of the 50 most-cited papers. The literature is dominated by journal articles and conference papers produced by small- to medium-sized research teams (mean: 3.9 authors per paper; h-index: 29). Keyword and overlay visualisations reveal four principal research axes: deep-learning-based gaze estimation; XR-related interaction paradigms within HCI; cognitive load and human factors; and usability- and accessibility-oriented interface design. The most-cited studies focus on gaze interaction in immersive environments, deep learning for gaze estimation, multimodal interaction, and physiological approaches to assessing cognitive load. Overall, the findings indicate that eye tracking in HCI is evolving from a measurement-oriented technique into a core enabling technology that supports interaction design, cognitive assessment, accessibility, and ethical considerations such as privacy. This review identifies research gaps and outlines future directions for benchmarking practices, real-world deployments, and privacy-preserving gaze analytics in HCI.

## 1. Introduction

Eye tracking technology has become increasingly central to HCI research by enabling detailed analysis of users’ visual attention dynamics. The human eye exhibits different types of eye movements, such as fixations and rapid movements (saccades); these movements provide direct information about users’ cognitive processes and the distribution of attention [1]. In particular, the declining cost of eye-tracking devices and their increasing portability in recent years have made this technology more widespread and accessible [2].

Early eye tracking studies in HCI aimed to go beyond traditional input devices such as the mouse and keyboard by using users’ gaze behaviour as a channel for data entry or interaction control. For example, the MAGIC (Manual and Gaze Input Cascaded) approach demonstrated the potential of combining eye movements with manual input [3]. Such approaches offer substantial advantages in terms of accessibility; for users with limited motor control, gaze-based interfaces can provide greater independence [4].

Eye tracking has also been positioned as a valuable method in user experience (UX) evaluation. By generating visual attention maps, it becomes possible to gain insights into which elements attract attention, which areas are overlooked, and users’ navigation strategies [5]. Integrating these insights into interface design and usability testing has enriched both the theoretical and applied dimensions of HCI research.

However, several essential challenges persist in eye tracking research conducted in HCI contexts. In particular, collecting and analysing data in experimental setups that more closely approximate real-world conditions (e.g., mobile devices; virtual reality (VR)/augmented reality (AR) environments) remains technically and methodologically complex [6]. On the other hand, machine-learning and AI-based gaze analysis methods offer new opportunities for estimating gaze direction and developing adaptive interfaces [7].

Unlike prior bibliometric or narrative overviews that focus on eye tracking within specific HCI subdomains (e.g., user experience, web usability, or automotive interfaces), this study provides a comprehensive mapping of eye-tracking research across the broader field of human–computer interaction from 2020 to 2025. By integrating records from WoS and Scopus, generating time-overlay keyword maps, and linking these maps to a qualitative synthesis of the most-cited papers, we identify the principal thematic clusters within HCI eye-tracking research and examine how eye tracking is operationalised both as a measurement tool and as an interaction technology.

The results reveal diverse application contexts, including XR-related interaction paradigms within HCI, deep-learning-based gaze estimation, cognitive load assessment, and usability and accessibility-oriented interface design. This dual perspective, capturing both topical structures and methodological roles, distinguishes our contribution from more narrowly focused HCI bibliometric studies and from purely experimental overviews that address eye tracking in isolated application domains.

In this paper, we systematically review the eye tracking and HCI literature and focus on the following research questions:

i. What are the quantitative characteristics of publications in eye tracking and HCI during the 2020–2025 period?
ii. What is the intellectual and thematic structure of this literature?
iii. How does the time-overlay keyword map reveal established and emerging research foci in eye tracking and HCI?
iv. Which application areas, methodological approaches, and future research directions are highlighted by the 50 most-cited studies in eye tracking and HCI?

To this end, we conducted a bibliometric analysis using publication data retrieved from the WoS and Scopus databases.

## 2. Related Work

The eye-tracking and HCI literature has undergone substantial methodological and technological transformations in recent years. Whereas early studies primarily focused on measuring visual attention in two-dimensional, screen-based settings, contemporary research has increasingly shifted toward analysing gaze behaviour in three-dimensional and virtual-reality settings. One of the most comprehensive reviews in this area is the work by Sundstedt and Garro, which systematically examines eye-movement data visualisation approaches for 3D environments and emphasises that next-generation eye-tracking research increasingly requires dynamic, multi-layered data analysis [8].

The use of gaze-based interaction applications within HCI has also expanded. Bozkir et al. provide an extensive review of gaze-aware virtual reality applications and show that structural issues, particularly privacy, biometric data security, and user consent, are now on the research agenda [9]. This indicates that not only technical accuracy but also ethical design principles have become critical for gaze-based systems.

In the gaze estimation literature, platform-dependent performance differences are particularly salient. Kar and Corcoran examine estimation algorithms used in consumer-grade devices and highlight the lack of standardisation in performance comparisons [10]. Similarly, Ghosh and colleagues compare deep-learning-based approaches and demonstrate that, in real-world usage scenarios, occlusion, head pose, and inter-individual differences in eye shape still lead to substantial performance degradation [7].

The rapid evolution of hardware platforms has also necessitated a re-examination of the relationship between measurement accuracy and user experience. For instance, an experimental study by Huang et al. on Apple Vision Pro shows that gaze-tracking accuracy influences user experience; however, user perception cannot be explained solely by measurement error [11]. This finding suggests that eye-tracking data should be evaluated not only on technical performance but also on perceived usability.

Eye tracking technology is also an essential tool for accessibility-oriented interaction systems. Taş and Yavuz developed a system that enables individuals with motor impairments to communicate in digital environments by leveraging eye, eyebrow, and head movements, demonstrating that this approach can serve as an alternative to traditional input devices [4]. Such work indicates that gaze-based control is relevant not only for cognitive research purposes but also for human-centred design principles.

Studies on appearance-based, real-time gaze estimation methods particularly aim to develop solutions with low hardware requirements. Yılmaz and Köse compare image-processing-based gaze-direction estimation methods and discuss their applicability to alternative input modalities in human–computer interaction [12]. The findings in this area suggest that a transitional phase is ongoing between classical image-processing approaches and deep-learning-based models in terms of performance.

Overall, the literature indicates that eye tracking research in HCI has developed along three principal axes: (i) the expansion of data analysis methods from 2D screens to 3D interactive environments; (ii) the rise of machine-learning-based gaze estimation models; and (iii) the growing prominence of accessibility, ethics, and privacy in the research agenda. These trends show that eye tracking technology has moved beyond being merely a measurement tool and has become a multifaceted research domain that bridges user experience, design, algorithmic trustworthiness, and cognitive modelling.

## 3. Materials and Methods

This study aims to examine the bibliometric characteristics of scientific publications on eye tracking and HCI published between 2020 and 2025. The research process consists of three main stages: (i) data collection, (ii) data merging and cleaning, and (iii) bibliometric and thematic analysis.

### 3.1. Data Sources and Search Strategy

Two international citation databases, WoS Core Collection and Scopus, were used in this study. Searches were conducted in both databases on 17 November 2025 using the Title, Abstract, and Keywords fields. The WoS search covered the Science Citation Index Expanded (SCI-EXPANDED), Social Sciences Citation Index (SSCI), Emerging Sources Citation Index (ESCI), and Conference Proceedings Citation Index–Science (CPCI-S). The Scopus search was performed across all subject areas. In the search strategy, terms representing eye tracking techniques and the HCI discipline were combined using Boolean operators. The query used was as follows:

(“eye tracking” OR “eyetracking” OR “gaze behavior” OR “visual attention”)

AND

(“human computer interaction” OR “HCI”)

This query was intended to capture scholarly publications that use eye movements in the context of human behaviour interacting with computer interfaces.

It should be noted that the export formats of WoS and Scopus do not provide device-level metadata (e.g., sampling rate, calibration procedures, spatial accuracy, data loss). Accordingly, our analyses focus on publication and topical structures rather than quantitative comparisons of eye-movement recording protocols.

### 3.2. Publication Inclusion Criteria

Only publications meeting the following criteria were included in the analysis:

- Publication years: 2020–2025
- Document type: Only records classified as article, conference paper, and conference review were included. Book chapters, editorials, short notes, letters, and similar document types were excluded because they typically do not report full empirical findings or provide comprehensive methodological descriptions, and they may distort citation dynamics in bibliometric analyses.
- Scope: Eye tracking and HCI
- Language: English
- Metadata completeness: Availability of complete information for title, author(s), year, source title, and DOI

These criteria were defined to ensure both conceptual alignment and data consistency.

### 3.3. Data Collection and Merging Procedure

The total number of publications retrieved from the databases is presented in Table 1.

Records obtained from WoS and Scopus were exported to CSV and merged using the “Merge Data” function in VOSviewer. Duplicate publications were identified and removed according to the following criteria: DOI match, exact title match, author order, and year match. After these procedures, 1180 duplicate records were removed, resulting in a final dataset of 1033 unique publications. This process was also documented using a PRISMA flow diagram.

### 3.4. Bibliometric and Thematic Analysis Procedure

VOSviewer (v.1.6.0) was used for the bibliometric analyses. Within the scope of the study, we generated a keyword co-occurrence map, a co-citation network, a co-authorship network, and a source (journal) interaction map. The resulting visualisations illustrate the thematic clusters of the research field, prominent concepts, and academic collaboration networks.

The abstracts of the 50 most-cited papers were qualitatively analysed by a single author using a structured coding protocol. An initial codebook was developed from a subset of documents and iteratively refined as new themes emerged. The final thematic structure includes categories such as “XR gaze interaction”, “deep-learning-based gaze estimation”, “cognitive load and physiological measures”, “usability and interface evaluation”, and “accessibility and assistive communication”. Because a single researcher conducted the coding, the thematic clustering should be interpreted as an informative yet necessarily interpretive synthesis rather than a consensus-based taxonomy.

### 3.5. PRISMA Flow Diagram

For publication-level analyses, we followed the core elements of the PRISMA-ScR extension, as applicable to bibliometric mapping studies (e.g., transparent reporting of the search strategy, selection criteria, and data extraction), while omitting formal risk-of-bias assessments, which are not standard for this type of analysis. The initial search was conducted on 17 November 2025 and was not updated, as the aim was to provide a time-bounded snapshot of the 2020–2025 period. After removing duplicates, titles and abstracts were screened against the inclusion criteria specified in Section 3.2 (eye tracking in an HCI context, document type, and language). Full texts were consulted only when relevance could not be determined from bibliographic metadata alone. A structured data-extraction sheet was used to record publication year, document type, venue, country, and citation counts for all included papers. Because our primary aim was to map the field’s structure rather than evaluate the effectiveness of specific interventions, we did not conduct formal risk-of-bias or methodological quality assessments of individual studies, consistent with standard practice in bibliometric and scoping reviews.

The purpose of the qualitative synthesis was to identify overarching thematic regularities in the most influential papers rather than to provide a detailed quantitative content analysis. Therefore, the single-coder procedure should be interpreted as an expert-driven, interpretive mapping exercise rather than a statistically validated coding scheme.

**Figure 1:**
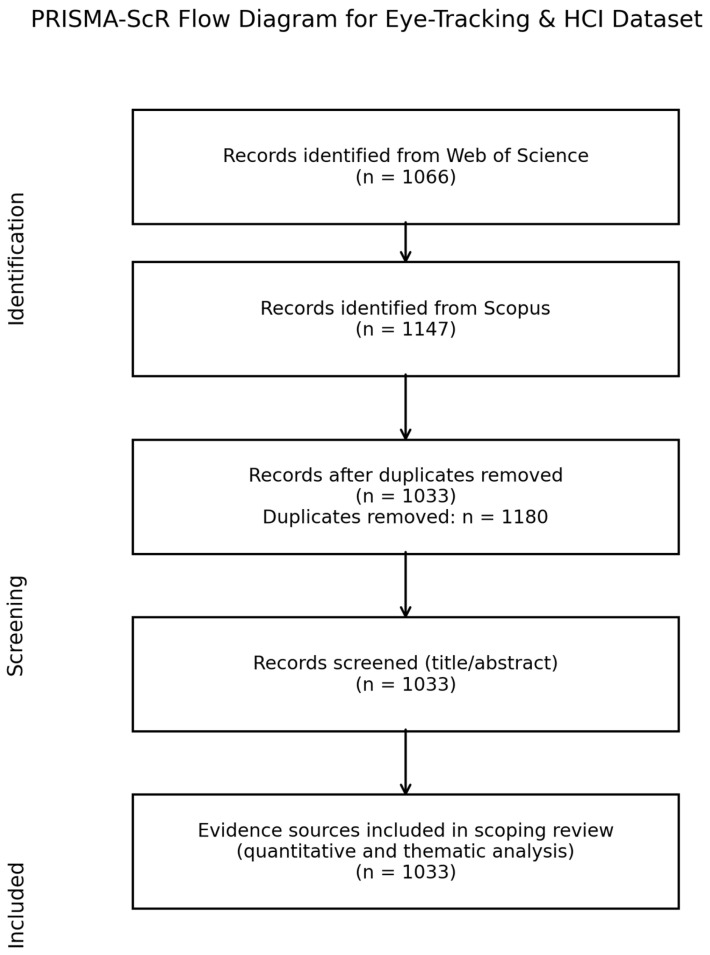
PRISMA flow diagram for eye tracking and HCI.

Data collection and cleaning procedures were reported in accordance with the PRISMA (Preferred Reporting Items for Systematic Reviews and Meta-Analyses) framework. The study flow diagram includes the steps shown in Figure 1.

As shown in Figure 1, a total of 1147 records were retrieved from Scopus and 1066 records from WoS, yielding 2213 raw records across the two databases. The records were then merged based on DOI information and title matching, and duplicate entries were removed. Following this matching and cleaning process, 1180 duplicate records were excluded, leaving unique publications. In the final stage, the dataset used for the bibliometric analyses comprised 1033 publications. This process is visually presented in the PRISMA flow diagram provided in the manuscript.

### 3.6. Quality Assessment and Handling of Discrepancies

Consistent with the bibliometric mapping approach, no formal risk-of-bias assessment of individual studies was conducted, as the aim was to map the field’s structure rather than evaluate intervention efficacy. However, to ensure data robustness and transparency, the following steps were taken during the merging and cleaning phase:

- Discrepancy Resolution: When merging records from WoS and Scopus, instances of conflicting metadata (e.g., publication year, author order, or journal title) were identified. For each conflict, the original publication was consulted via its DOI or through a direct database search. The record with the most complete and accurate information was retained in the final dataset.
- Duplicate Removal Protocol: Duplicates were removed using a hierarchical matching criterion: (i) exact DOI match, (ii) exact title match (case-insensitive), (iii) matching first author and publication year. This process was documented in a log file (available upon request).
- Methodological Transparency: While bibliometric metadata does not encode experimental details (e.g., eye-tracker calibration, sampling rate), we acknowledge this as a limitation of such analyses. Our focus remains on publication and thematic trends, not on methodological benchmarking.

### 3.7. Use of Generative AI Tools

During the preparation of this manuscript, a generative AI tool (ChatGPT 5.2, OpenAI) was used solely to assist with language editing and stylistic refinement, including improving clarity, coherence, and academic tone of the English text. The tool was not used for data collection, data analysis, result interpretation, or figure generation. All outputs were carefully reviewed and edited by the authors, who take full responsibility for the content of this publication.

## 4. Results

The merged Scopus–WoS dataset comprises 1033 publications authored by 3359 unique contributors. The mean number of authors per publication is 3.9 (approximately 4), with a median of 4 (range: 1–18). Approximately 6% of the publications are single-authored, 18% involve two authors, and 44% include three or four authors. Although papers with larger author teams are less common, multi-authored publications (five or more authors) indicate large-scale experimental studies and multi-centre collaborations. Overall, these findings indicate that research in eye tracking and HCI is predominantly produced by small- to medium-sized teams. At the same time, broader collaborative efforts play a role in specific research contexts.

Regarding publication venues, the vast majority of the 1033 records are conference papers and journal articles. Conference papers account for approximately 620 records, followed by journal articles with around 320 publications. Together, these two formats represent nearly the entire body of literature, underscoring the central role of conferences alongside peer-reviewed journals in disseminating eye-tracking and HCI research, particularly for the rapid communication of experimental findings.

By contrast, review articles (n ≈ 20) and other document types (e.g., editorials, notes, or early-access items; n ≈ 70) constitute only a small proportion of the total output. This distribution suggests that the literature is largely experiment-driven, with relatively limited emphasis on integrative or theory-building reviews. In other words, the field appears to prioritise the presentation of new empirical results, while synthesis-oriented review work remains comparatively under-represented.

### 4.1. Keyword Co-Occurrence Map

**Figure 2:**
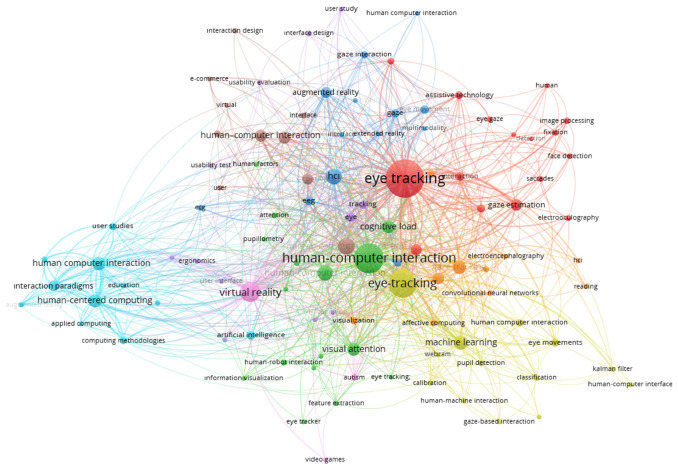
Keyword co-occurrence network of eye tracking and HCI publications (2020–2025) (VOSviewer; all author keywords; minimum three occurrences).

Using VOSviewer, a co-occurrence map was generated based on the author keywords of the 1033 publications in the merged dataset. The map was produced at the level of all author keywords using the full-counting method, with a threshold of at least three occurrences per keyword. A total of 104 keywords meeting this threshold were clustered and visualised (Figure 2).

In the centre of the map in Figure 2, the node “eye tracking” is the most dominant concept, both in node size and in the number of links, indicating that eye tracking is positioned as a shared core technology that connects all other research axes. The presence of directly connected nodes such as “visual attention”, “virtual reality”, “usability”, “gaze tracking”, “cognitive load”, “deep learning”, “computer vision”, and “human-centered computing” suggests that technical developments and human-centred/cognitive dimensions are closely intertwined in the field.

An understanding of the colour-coded clusters indicates that the literature is organised around several dominant themes:

Technical/algorithmic cluster (e.g., green-toned cluster): This cluster, characterised by terms such as “gaze estimation”, “deep learning”, “computer vision”, “image processing”, “xgboost”, and “multimodal interaction”, represents studies focusing on gaze estimation, deep-learning models, and computer-vision-based eye tracking algorithms. Work in this area particularly concentrates on real-time gaze tracking and improving model accuracy.

XR and interaction-paradigm cluster (e.g., orange/brown-toned cluster): Keywords such as “virtual reality”, “augmented reality”, “interaction paradigms”, “human-centered computing”, “emotion recognition”, “convolutional neural network”, and “serious game” bring together studies in which eye tracking is used in VR/AR environments as an interaction technique and for purposes such as gamification and emotion recognition. This cluster indicates that eye tracking is used not only as a measurement tool but also as an interaction paradigm.

Cognitive load and human-factors cluster (e.g., blue-toned cluster): Concepts including “cognitive load”, “human factors”, “attention”, “mental workload”, “working memory”, “reading”, and “supervisory control” point to research that uses eye movements to assess cognitive processes, workload, and performance. These studies are particularly concentrated in contexts such as education, supervisory tasks, and interaction with complex interfaces.

Usability and interface-design cluster (e.g., pink/purple-toned cluster): The grouping of terms such as “usability”, “interface design”, “input devices”, “accessibility”, “extended reality (XR)”, “digital twin”, “pupil size”, and “saccades” shows that eye tracking is used as a systematic metric in interface design, interaction techniques, and accessibility evaluations. This cluster represents the literature in which classic HCI usability studies are enriched with eye-tracking measures.

### 4.2. Co-Authorship Network

**Figure 3:**
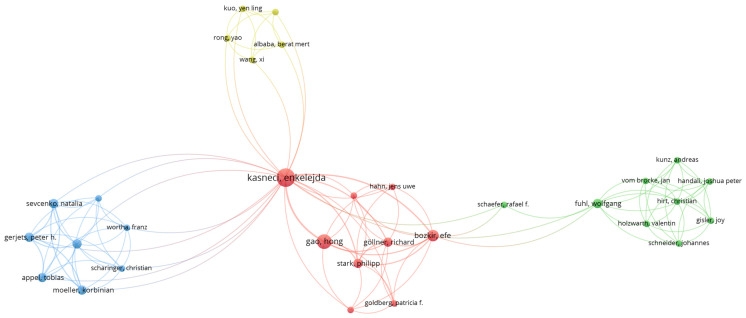
Co-authorship network in eye tracking and HCI (VOSviewer; authors with at least one publication and one citation).

The co-authorship analysis illustrates the field’s productive and central researchers and the collaboration patterns among them. The study was conducted using the author’s unit and was restricted to researchers who had co-authored at least three publications. The resulting network, consisting of 54 researchers who meet this threshold, splits into several significant components (Figure 3).

The most densely connected centres of the network are clustered around research groups based in Europe (particularly Germany and Finland) and Asia (China and South Korea). Groups working on XR and wearable devices, as well as those focusing on usability and UX, appear to be partially separated in terms of co-authorship. This pattern suggests that technological development-oriented work and applied HCI research are, to some extent, conducted by different scholarly communities.

Figure 3 illustrates the co-authorship structure of eye-tracking research in human–computer interaction at the cluster level. Rather than visualising all individual authors who met the inclusion threshold simultaneously, the network is presented in a simplified form to preserve interpretability and to emphasise the global collaboration structure. In the figure, nodes represent authors and edges indicate co-authorship relations; node size reflects relative publication productivity, and edge thickness reflects the strength of collaborative ties.

The resulting clustering reveals a research landscape characterised by several medium-sized, densely connected collaboration groups, alongside a smaller number of highly central authors who act as bridges between otherwise weakly connected clusters. These bridging authors occupy structurally important positions, facilitating information flow and intellectual exchange across different thematic or institutional groups.

This configuration suggests that eye-tracking research in HCI is organised around multiple semi-autonomous collaboration units, often aligned with specific experimental paradigms, application domains, or research traditions. At the same time, the presence of cross-cluster connections indicates a mature and integrated research field in which collaboration extends beyond isolated teams. Overall, the co-authorship network highlights a balance between localised, team-based knowledge production and broader field-level collaboration dynamics that support specialisation, methodological diversity, and interdisciplinary interaction.

### 4.3. Citation Analysis: Most-Cited Studies and Sources

Across the 1033 publications in the merged Scopus–WoS dataset, the total number of citations was 4407. The mean number of citations per publication is 4.27, while the median is 0, indicating that a relatively small number of highly cited papers drive citation impact in the field. In contrast, a substantial share of publications is still in the early stages of citation accumulation. The dataset has an h-index of 29, meaning that 29 publications have received at least 29 citations. The most-cited publication has 151 citations, while the majority of records have 10 or fewer citations.

When the most-cited publications are examined in Table 2, it is evident that the majority of the top 10 studies are broad reviews or surveys. In particular, themes such as the use of eye tracking in extended/mixed reality (XR) environments, deep-learning-based gaze estimation, the recognition of eye expressions and short-term affect, and multimodal interaction (e.g., gaze, gestures, speech) emerge prominently. This pattern confirms that the contemporary research agenda in the field is concentrated around both XR and novel interaction paradigms, as well as AI and deep-learning-based analytical methods.

From a source level perspective, the outlets with the highest number of publications include the Lecture Notes in Computer Science (LNCS) and Communications in Computer and Information Science series, as well as open-access journals such as IEEE Access, Sensors, and Applied Sciences (Switzerland). Among venues more specific to HCI and eye tracking, the Conference on Human Factors in Computing Systems (CHI), the Proceedings of the ACM on Human–Computer Interaction, and the ACM Symposium on Eye Tracking Research and Applications (ETRA) are particularly notable. Although CHI and related HCI conference series publish fewer papers, their higher citations per paper indicate that they are core outlets with strong influence in the field.

Overall, these findings show that eye tracking and HCI research are concentrated in both computer-science-oriented conference series and open-access, multidisciplinary journals. They further indicate that highly cited contributions are predominantly survey papers summarising the state of the art, alongside pioneering studies focusing on emerging interaction technologies (e.g., XR, deep-learning-based gaze estimation).

While co-authorship and co-citation networks help identify influential research groups, venues, and intellectual lineages, their explanatory power with respect to experimental eye-movement methodology is limited. Such networks primarily visualise how authors and publications are connected through co-publication patterns and shared references, and, as a result, they are well-suited to mapping the social and intellectual structure of a research field. However, they do not directly encode core methodological decisions that are critical in eye-movement research, such as experimental paradigms, calibration procedures, sampling rates, data-quality criteria (e.g., data loss), or event-detection algorithms. Consequently, the patterns observed in these networks should not be interpreted as a direct proxy for methodological convergence or best practice in experimental eye tracking. Therefore, the networks reported here provide complementary context that situates eye tracking studies within broader HCI/XR collaboration patterns and intellectual structures, rather than a methodological characterisation of experimental design choices or data-processing pipelines. In this sense, the network maps are intended to support the interpretation of the field’s development and connectivity. At the same time, detailed methodological comparisons remain outside the scope of bibliometric mapping based solely on bibliographic metadata.

### 4.4. Time-Overlay Keyword Analysis (Time-Overlay Keyword Map)

**Figure 4:**
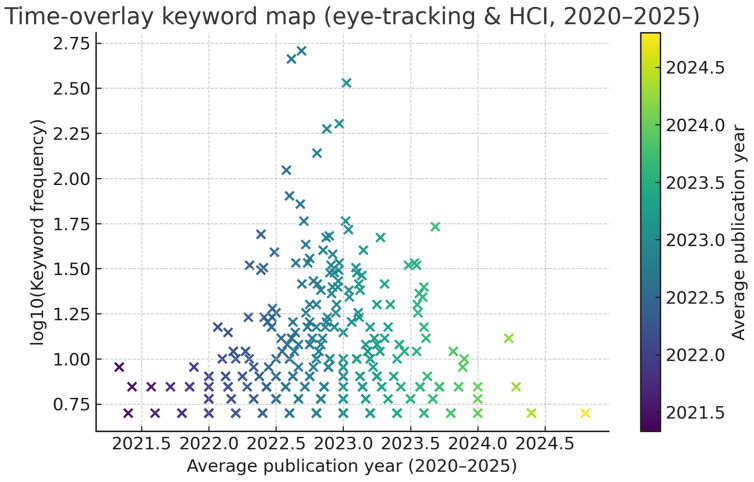
Time-overlay keyword map (2020–2025).

For 361 keywords that occurred at least 5 times during the 2020–2025 period, the average publication year for each term was calculated, and a time-overlay keyword map was generated accordingly (Figure 4). This approach visualises both established themes and emerging research areas that have gained prominence in recent years.

The results shown in Figure 4 indicate that the eye tracking and HCI literature can be divided into three main phases over time:

- Early-phase themes (around 2021): Keywords with an average publication year of approximately 2021 include “surveys”, “information visualization”, “human–machine interface”, “predictive analytics”, “pupillometry”, “e-commerce”, and “data collection”. This period represents a phase in which eye tracking was primarily associated with classic HMI/HCI contexts and data collection–measurement techniques, and in which literature reviews and state-of-the-art contributions were particularly prevalent.
- Transitional-phase themes (2022–2023): Terms with average year values in the 2022–2023 range suggest that eye tracking expanded toward application domains such as medical computing, wearable technology, navigation, education, and cognitive performance measurement. In this phase, eye tracking was most often used as a measurement tool to enrich existing HCI scenarios.
- Emerging themes (late 2023–2024): On the rightmost side of the map are the most recent keywords with an average year of 2023.8 and above. These include advanced machine-learning approaches such as “contrastive learning” and “adversarial machine learning”; interaction technologies related to gaze-based control such as “eye controlled devices” and “cursor control”; and concepts associated with XR and interaction contexts such as “virtual environments”, “reality” (in extended/virtual reality contexts), “interaction”, “human–computer”, “eye-movements”, “emotions”, and “augmentative and alternative communication”. This clustering indicates that, in recent years, eye tracking research has shifted toward interaction in XR environments, gaze-controlled devices and accessibility (particularly for users with speech or motor limitations), affective and behavioural recognition, and advanced modelling approaches enriched with contrastive and adversarial learning.

Overall, the time-overlay keyword map reveals a clear thematic shift in the eye tracking and HCI literature: an early emphasis on core methods and survey-oriented accumulation in 2020–2021, diversification of application domains in 2022–2023, and increasing prominence of XR, advanced machine learning, and gaze-controlled interaction devices in late 2023 and 2024.

### 4.5. Thematic Content Analysis of the 50 Most-Cited Publications

To complement the bibliometric mapping, a qualitative content analysis was conducted on the 50 most-cited publications in the dataset. These publications were issued between 2021 and 2024 and received a total of 1718 citations, averaging 34.4 per paper. The studies span a wide range of areas, including eye tracking technologies, XR, HCI, medical and educational applications, novel hardware, and privacy concerns.

#### 4.5.1. Main Themes

- XR and Gaze-Based Interaction

The most dominant theme concerns the integration of eye tracking into XR environments. The most-cited review provides a comprehensive synthesis of gaze interaction and eye tracking research in XR, serving as a foundational reference for the field [13]. The studies show that eye tracking in XR systems is used both as an input for interaction and as an analytical tool for modelling user behaviour [14]. In particular, in remote collaboration scenarios, sharing gaze information improves communication quality and task performance, and different gaze-visualisation strategies have notable effects on symmetric collaboration and joint attention [15,16,17].

- Deep Learning and Gaze Estimation

The second theme focuses on gaze estimation and eye-movement analysis using machine learning and deep learning methods. Convolutional neural networks and related architectures have been shown to improve accuracy and robustness, particularly under unconstrained viewing conditions [7,18]. These approaches enable automated classification of eye movements and real-time gaze estimation in interactive systems; novel sensing channels such as event-based cameras and multi-task face analytics further enrich gaze and facial-expression analysis with greater temporal precision [19,20,21].

- Medical and Clinical Applications

The third theme addresses the use of eye tracking in medical and clinical contexts. Robotic nurse assistants that coordinate their behaviour based on surgeons’ gaze during surgical procedures [22,23] and eye-tracking systems integrated with digital-twin models in skull-base surgery [24] provide illustrative examples of clinical decision support and training. VR systems that facilitate joint-attention practice for children with autism spectrum disorder [25] and affective VR experiences for older adults with cognitive impairment [26] demonstrate the potential of eye tracking for rehabilitation-oriented applications.

- Cognitive Load and Mental-State Assessment

The fourth theme encompasses the use of eye tracking, along with other physiological indicators, to estimate cognitive load, mental workload, and affective state. Physiological methods for measuring cognitive load in augmented reality have been systematically reviewed [27], and cognitive load levels in emergency simulation games have been classified using machine learning [19]. In aviation, real-time task-load indices based on gaze and multiple physiological signals have been proposed [28]. In short-term affect recognition, classifications obtained from spatiotemporal EEG modelling are behaviourally supported by eye-tracking data [29].

- Educational and Learning Environments

The fifth theme covers the use of eye tracking in educational contexts. Key examples include analyses of students’ attention patterns in VR classrooms through salient objects [30] and work conceptualising Learning Experience Design (LxD) using learner data derived from eye tracking [31]. Visual search and information-selection strategies in online information problem-solving [32], as well as machine-learning-supported eye tracking in interactive geographic visualisation [33], reflect a broader trend toward learning analytics and adaptive educational technologies.

- Multimodal Interaction Systems

The sixth theme concerns the integration of eye tracking into multimodal interaction architectures. Flexible AR systems that combine gaze, gestures, and speech [34] and mapping studies of sensor–AI methods indicate that eye tracking is increasingly embedded within broader multi-sensor ecosystems [35]. Application examples include context-aware voice assistants in VR that use gaze points to resolve pronominal ambiguity [36] and gaze-aware information access in AR that provides advantages in hands-busy tasks [37].

- Privacy and Security

Given the personal and behavioural nature of gaze data, privacy-oriented research has gained visibility. Differential-privacy approaches proposed for temporally correlated eye-tracking data aim to reduce re-identification risk while preserving analytic utility, underscoring the importance of privacy-aware design in eye-tracking systems [38].

- Industrial and Commercial Applications

In industrial settings, studies examine how eye tracking in AR-supported workflows affects task time, error rates, and mental workload [39]. In commercial contexts, the effects of recommender systems on purchasing behaviour have been analysed using gaze and event-tracking data [40]. Eye tracking has also been used to evaluate multimodal interaction designs in museums and art spaces [41] and to assess factors shaping viewing behaviour on search-engine results pages [42].

- Games and Entertainment

This theme addresses the use of eye tracking in video games. Representative studies include investigations of how different interaction modalities affect arousal and engagement [43]; the use of eye tracking as a tool for monitoring player state in dynamic difficulty adjustment [44]; analyses of visual behaviour and performance among expert action-game players [45]; and reviews focusing on biofeedback and physiological interaction techniques in entertainment games [46].

- Emerging Technologies and Devices

The final theme includes novel hardware and sensing technologies. Examples of hardware innovation include transparent, flexible, and ultra-stable electrostatic interfaces [47] and wearable eye tracking solutions developed using graphene-based textile materials [48]. Validation studies of low-cost eye-tracking and psychophysiology toolkits [49] and reviews examining eye-tracking technologies that leverage edge computing on mobile devices [50] discuss resource constraints, latency, and privacy-related considerations.

#### 4.5.2. Methodological Approaches

Across the 50 most-cited papers, the methodological approaches can be grouped into several main categories:

- Experimental user studies: Controlled experiments, usability testing, and performance evaluations conducted in XR, gaming, industrial, and educational scenarios represent the most common approach [39,43,44].
- Systematic literature reviews and mapping studies: These studies synthesise accumulated knowledge on topics such as multimodal sensing, dynamic difficulty adjustment, and edge-based eye tracking [36,44,50].
- Machine-learning-based models: Deep neural networks, classifiers, and spatiotemporal models are widely used for gaze estimation, classification of cognitive/affective states, and analysis of complex behavioural patterns [18,19,29].
- Physiological measurements: Eye movements, EEG, heart rate, and other biometric signals enable multi-indicator assessment of cognitive load, workload, and affect [27,28,39].
- System design and prototyping: This category includes studies proposing novel interfaces and interaction techniques that integrate eye tracking into platforms such as XR, medical training, remote collaboration tools, and multimodal assistants [24,25,34,36].

#### 4.5.3. Research Trends and Future Directions

The thematic and methodological patterns observed in the 50 most-cited publications point to several key trends:

- Shift toward multimodal systems: Eye tracking is increasingly considered together with speech, gestures, physiological signals, and contextual information, becoming a central component of rich multimodal interaction frameworks.
- Real-time processing and adaptive interaction: Systems are increasingly oriented toward on-the-fly analysis and feedback, enabling dynamic adaptation in games, time-critical support in aviation and emergency scenarios, and real-time guidance in surgery.
- Tight integration with AI: Deep, adversarial, and contrastive learning techniques are being integrated into gaze estimation and user-behaviour modelling to develop more robust and generalisable eye tracking systems.
- Privacy-aware design: Given the sensitivity of gaze data, privacy-preserving approaches such as differential privacy, data minimisation, and local/edge computing are gaining importance.
- Domain-specific applications: Tailored solutions are being developed for sectors such as medicine, education, industry, and entertainment, with attention to accuracy, latency, interpretability, and user acceptance.
- Remote collaboration and work environments: In the post-pandemic period, eye tracking has become a key component of remote work, telepresence, and collaborative XR tools, and the volume of research in this area has increased markedly.

Overall, this content analysis indicates that eye tracking research in HCI/XR is interdisciplinary and rapidly evolving. Advances in hardware, AI, and immersive technologies are making eye-tracking systems more accurate, accessible, and tightly integrated with interactions. The findings suggest that eye tracking will be increasingly embedded in everyday contexts—from clinical practice to educational technologies, industrial production, and games and entertainment—and that human–machine interaction will become more natural, adaptive, and context-aware.

## 5. Discussion

### 5.1. Quantitative Characteristics of Publications (RQ1)

This study examined 1033 publications on eye tracking in human–computer interaction (HCI) published between 2020 and 2025, revealing the field’s structural characteristics and thematic trajectories. The findings suggest that the literature appears to be maturing/appears mature in both volume and content, while also undergoing a pronounced transformation driven by emerging technologies and expanding application domains. The dominance of journal articles and conference papers indicates that experimental and applied studies largely shape progress in this area. In contrast, review and theoretical contributions constitute a comparatively smaller share. This publication profile aligns with prior work that has framed eye tracking as a methodological instrument in HCI contexts [1,2,5].

### 5.2. Thematic Structure and Keyword Evolution (RQ4)

The keyword co-occurrence analysis and the time-overlay map suggest that eye tracking research clustered around four principal axes: (i) deep learning and algorithmic gaze estimation, (ii) extended reality (XR) and novel interaction paradigms, (iii) cognitive load and human factors, and (iv) usability- and accessibility-oriented interface design. This thematic structure quantitatively corroborates trends highlighted in systematic reviews focusing on eye-movement data visualisation in 3D environments [8] and comprehensive examinations of eye-tracked virtual reality applications [9]. The increasing frequency and later average publication years of XR-related keywords further align with Plopski et al.’s review of gaze interaction in XR, suggesting that the centre of gravity of the field is shifting toward head-mounted XR devices and immersive interaction environments [13].

### 5.3. High-Impact Publications and Intellectual Structure (RQ5)

The citation and content analyses of the 50 most-cited publications show that these thematic clusters are not only prevalent but also influential. A substantial proportion of the most-cited studies comprises reviews or methodological papers addressing gaze interaction in XR [14,15,16,17,18], deep-learning-based gaze estimation [18,19,20], and cognitive load and affect recognition supported by physiological signals [27,28,29]. This pattern suggests that survey- and methodology-focused work serves as an “intellectual backbone” that shapes the knowledge infrastructure of eye-tracking and HCI research.

### 5.4. Application Domains and Use Contexts

Beyond these dominant clusters, the content analysis indicates that studies in medical/clinical applications, educational and learning settings, and games and entertainment also achieve high citation impact [21,22,23,24,25,26,31,32,33,34,44,45,46,47]. Robotic nurse assistants that coordinate behaviour based on surgeons’ gaze in surgical workflows [22] and digital twin-based systems integrated into skull-base surgery [24] exemplify how eye tracking can be operationalised for clinical decision support and training. VR systems that facilitate joint-attention practice for children with autism spectrum disorder [25] and affective VR experiences designed for older adults with cognitive impairments [26] further demonstrate the growing presence of eye tracking in rehabilitation- and quality-of-life-oriented applications. In education and learning research, approaches such as attention analysis in VR classrooms [30] and learning experience design [29] point to an emerging line of work in which eye tracking is increasingly integrated with learning analytics.

### 5.5. Methodological Implications for Eye-Movement Research

Multimodal interaction systems and privacy-oriented research represent critical junctions for the field’s future development. AR systems that combine gaze, gesture, and speech [34], together with systematic mapping studies of sensor and AI methods [35], indicate that eye tracking is becoming embedded in richer multi-sensing architectures. Conversely, differential-privacy methods proposed for gaze data across contexts [38] and the privacy risks discussed in gaze-aware VR applications [9] suggest that ethical and legal dimensions are becoming increasingly prominent as eye-tracking technologies diffuse into everyday use.

Compared with prior bibliometric reviews that are narrower in scope or focused on specific subdomains, this study aims to offer three main contributions. First, by jointly using WoS and Scopus, it provides a comprehensive cross-section of eye-tracking–HCI for 2020–2025, thereby partially mitigating potential domain bias associated with single-database studies [5,6]. Second, by combining bibliometric network analyses (keyword co-occurrence, co-citation, and co-authorship) with qualitative content analysis, the study demonstrates not only the existence of thematic clusters but also how these clusters are represented in high-impact publications. Third, through time-overlay keyword analysis, it provides quantitative evidence of a shift from survey- and method-oriented accumulation toward XR, AI, and multimodal interaction within the eye-tracking and HCI literature.

This study also speaks directly to methodological discussions in eye-movement research. The inclusion of specialised, method-focused outlets within the dataset, particularly publications addressing measurement design, data-collection protocols, and analytic methods, highlights the importance of dedicated methodological venues in shaping and disseminating best practice. Building on this body of work, our findings show (i) which interaction scenarios eye tracking research concentrates on, (ii) which measurement and analysis approaches are associated with higher citation impact, and (iii) how gaze data are positioned in XR, multimodal interaction, and cognitive assessment contexts.

Notably, the inclusion of work such as GaVe, which focuses on the visualisation and comparative analysis of eye-movement data [51], further illustrates the contribution of dedicated methodological research to the development and standardisation of eye-tracking practice.

Although our database does not encode eye-movement metrics as structured metadata, the qualitative examination of the 50 most-cited papers provides an informative indication of which measures are most central in current HCI/XR studies. In highly cited work, fixation-based measures (e.g., mean fixation duration, fixation count, fixation counts within areas of interest), saccade-related measures (e.g., saccade amplitude and frequency), and pupil-based indices (e.g., pupil dilation as an indicator of mental effort) are commonly used as primary outcomes. By contrast, more complex constructs such as scanpath entropy, transition matrices, or gaze deviation from optimal expert models appear less frequently. In many XR and gaming scenarios, eye tracking is combined with additional physiological channels (e.g., EEG, heart-rate variability, galvanic skin response) to derive composite indices of cognitive load or affective state. In usability and accessibility studies, gaze measures are used primarily to localise breakdowns in interaction flows and to assess visual search efficiency.

These patterns imply at least three methodological considerations for experimental research. First, in immersive XR settings, metric selection should be explicitly justified, for example, by clarifying whether fixation duration is interpreted as an index of visual processing, cognitive load, or uncertainty. Second, the frequent combination of gaze with additional physiological and behavioural signals highlights the value of multimodal designs that can disambiguate whether changes in gaze dynamics reflect perceptual, cognitive, or affective processes. Third, transparent reporting of data-processing procedures, including event-detection algorithms, parameter settings, and exclusion criteria, is essential for reproducibility and meaningful comparison across laboratories and device platforms.

### 5.6. Limitations and Interpretive Scope

At the same time, our mapping results point to persistent methodological gaps in the eye-movement literature. Many studies provide limited detail regarding eye-tracker calibration protocols, measurement accuracy, and data-loss rates. In addition, the classification and analysis of eye-movement types (e.g., saccades, fixations, smooth pursuit) and pupil-based measures (pupil diameter, pupil dynamics) are often not standardised. This lack of standardisation reduces comparability across experimental setups and device platforms. It indicates the need for more transparent, harmonised reporting standards for device calibration, data quality, and the operational definitions of eye-movement events.

This study has several significant limitations that should be considered when interpreting the findings. First, the analyses rely on a keyword-based search strategy. Because the query was restricted to terms such as “eye tracking”, “gaze estimation”, and “human–computer interaction”, some relevant studies may have been missed if they emphasised alternative terminology in titles, abstracts, or keywords (e.g., “gaze-based interaction”, “oculomotor behaviour”). This concern is particularly relevant to peripheral application areas where eye tracking is used as a secondary instrument. To mitigate this risk, we combined two major citation databases and complemented keyword-based mapping with a qualitative reading of the 50 most-cited papers; however, some degree of conceptual undercoverage remains possible. Second, WoS and Scopus export formats do not include device-level methodological metadata (e.g., tracker type, sampling rate, calibration procedure, spatial accuracy, data-loss rates). As a result, the bibliometric maps cannot differentiate among hardware platforms, display configurations, or calibration protocols, nor can they support meta-analytic comparisons of core eye-movement metrics (e.g., fixation-duration thresholds, saccade-detection algorithms, pupil-size preprocessing, scanpath metrics). Accordingly, the results should be interpreted as a publication- and topic-level cartography of eye-tracking research in human–computer interaction (HCI), rather than as a quantitative meta-analysis of eye-movement methodology. We explicitly avoid claims about optimal sampling frequencies, fixation definitions, or data-processing pipelines. Instead, we treat the absence of standardised methodological metadata as a significant research gap and argue that future studies would benefit from more systematic reporting of device characteristics, calibration procedures, metric definitions, and data-quality indicators.

As in any qualitative coding effort, the themes identified here reflect an interpretive synthesis and should be read as a plausible structuring of the literature rather than the only possible classification. Moreover, rather than attempting to cover the entirety of eye-movement research, our focus is on how eye tracking is situated within the broader field of human–computer interaction, particularly in applied interaction contexts that have expanded rapidly in recent years. Within this broader HCI landscape, XR-related studies emerge as a prominent application domain, reflecting the growing portfolio of immersive and gaze-based interaction research.

### 5.7. Future Directions

Several directions for future work follow these findings. First, benchmark studies based on open datasets are needed to systematically compare gaze-estimation algorithms across different hardware and software platforms [7,10,18,19,20]. Second, comprehensive experimental studies that jointly examine eye-tracking accuracy in XR and wearable contexts, as well as user experience and perceived reliability, remain limited [11,13,14]. Third, it is essential to embed privacy- and consent-sensitive design principles into both data-collection protocols and real-time interactive systems [9,38]. Fourth, beyond short laboratory-based experiments, longitudinal eye-tracking studies examining naturalistic usage scenarios and long-term behavioural patterns could provide richer insights for HCI design. Finally, analysing gaze data alongside other behavioural and neurophysiological indicators using explainable and interpretable AI models may contribute to both the development of cognitive models and increased user trust.

## 6. Conclusions

This study provides a comprehensive bibliometric and thematic overview of eye-tracking research in human–computer interaction (HCI), based on 1033 publications from 2020 to 2025. By integrating records from WoS and Scopus and combining network-based bibliometric analyses with a qualitative synthesis of highly cited studies, the review offers a structured account of how eye tracking is currently positioned within the HCI research landscape.

At a high level, the findings indicate that contemporary eye-tracking research in HCI is organised around several dominant thematic directions, including deep-learning-based gaze estimation, XR-related interaction paradigms, cognitive and human factors, and usability- and accessibility-oriented design. Collectively, these themes suggest a shift in the role of eye tracking from a primarily measurement-oriented technique toward a broader enabling technology that supports interaction design, cognitive assessment, and user-centred system development.

A key contribution of this study lies in clarifying the evolving methodological and application-oriented roles of eye tracking within HCI, while situating XR as a prominent, rapidly emerging application domain rather than a defining boundary of the field. The results further highlight the growing importance of methodological robustness, including the need for standardised evaluation protocols, benchmark datasets, and privacy-aware approaches to gaze data analysis.

Looking ahead, future research would benefit from more integrative and interdisciplinary approaches that connect eye tracking with advances in artificial intelligence, multimodal interaction, and real-world deployments. In particular, studies that move beyond controlled laboratory settings toward ecologically valid and ethically grounded applications are likely to play a crucial role in shaping the next phase of eye-tracking research in HCI. Overall, this review provides a consolidated reference point for researchers seeking to understand current trends and to identify strategic directions for future work.

## Figures and Tables

**Table 1:** Publication counts.

Database	Number of Publications
WoS	1066
Scopus	1147
Total (raw data)	2213

**Table 2:** Top 10 most-cited documents in the dataset.

Authors	Article Title	Journal/Source Title	Year	Cited by
Plopski, A.; Hirzle, T.; Norouzi, N.; Qian, L.; Bruder, G.; Langlotz, T.	The Eye in Extended Reality: A Survey on Gaze Interaction and Eye Tracking in XR	ACM Computing Surveys	2023	151
Ratclife, J.; Soave, F.; Bryan-Kinns, N.; Tokarchuk, L.; Farkhatdinov, I.	Extended reality (XR) remote research: A survey of tools, methods, and challenges	Conference on Human Factors in Computing Systems (CHI)	2021	132
Shi, Y.; Yang, P.; Lei, R.; Liu, Z.; Dong, X.; Tao, X.; Chu, X.; Wang, Z.; Chen, X.	Eye tracking and eye expression decoding based on transparent, flexible and ultra-persistent electrostatic interface	Nature Communications	2023	111
Tan, C.; Šarlija, M.; Kasabov, N.	NeuroSense: Short-term emotion recognition and understanding based on spiking neural network modelling of spatio-temporal EEG patterns	Neurocomputing	2021	97
Pathirana, P.; Senarath, S.; Meedeniya, D.; Jayalal, D.	Eye gaze estimation: A survey on deep learning-based approaches	Expert Systems with Applications	2022	81
Kwak, Y.; Song, W.-J.; Kim, S.-E.	FGANet: fNIRS-guided attention network for hybrid EEG-fNIRS brain-computer interfaces	IEEE Transactions on Neural Systems and Rehabilitation Engineering	2022	77
Wang, Z.; Wang, H.; Yu, H.; Lu, F.	Interaction with gaze, gesture, and speech in extended reality	IEEE Transactions on Human-Machine Systems	2021	54
Šumak, B.; Brdnik, S.; Pušnik, M.	Sensors and artificial intelligence methods and applications in extended reality systems and environments	Sensors	2022	52
Drouot, M.; Le Bigot, N.; Bricard, E.; De Bougrenet de la Tocnaye, J.-L.; Nourrit, V.	Augmented reality on industrial assembly line: eye tracking for cognitive workload assessment	Applied Ergonomics	2022	51
Pustejovsky, J.; Krishnaswamy, N.	Embodied Human Computer Interaction	KI—Künstliche Intelligenz	2021	51

## Data Availability

No new data were created or analyzed in this study. Data sharing is not applicable to this article.

## Abbreviations

The following abbreviations are used in this manuscript:

WOS	Web of Science
HCI	Human Computer Interaction
XR	Extended Reality
VR/AR	Virtual Reality/Augmented Reality
